# Physical and Numerical Simulations on Mechanical Properties of a Prefabricated Underground Utility Tunnel

**DOI:** 10.3390/ma15062276

**Published:** 2022-03-19

**Authors:** Yachuan Kuang, Zhiwei Peng, Jiahui Yang, Miaomiao Zhou, Chang He, Yinhu Liu, Xiaofei Mo, Zhexuan Song

**Affiliations:** 1 School of Civil Engineering, Central South University, Changsha 410075, China; kuangyachuan@csu.edu.cn (Y.K.); peng_zhiwei111@163.com (Z.P.); yangjiahui0930@163.com (J.Y.); songzhexuan@longfor.com (Z.S.); 2 Zhejiang Herun Tiancheng Real Estate Co., Ltd., Hangzhou 310000, China; 214811124@csu.edu.cn; 3 Powerchina Zhongnan Engineering Co., Ltd., Changsha 410014, China; 19307481473@sina.cn; 4 Jinke Property Group Co., Ltd., Chongqing 400020, China; csutmy@163.com

**Keywords:** prefabricated, underground utility tunnel, U-shaped ferrule joint bar connections, full-scale model tests, mechanical properties, numerical analyses

## Abstract

The “U-shaped ferrule joint bars connections” have a stable mechanical property, requiring a low level of construction accuracy and a relatively simple connection process, which significantly increase the construction speed. Based on the “U-shaped ferrule joint bars connections” technology, a new type of prefabricated concrete underground utility tunnel was proposed. This prefabricated technology realizes a formwork-free construction and vertical support-free assembly of the top plate on site. Through the full-scale model static test and numerical analyses, the mechanical properties, i.e., the crack development law and bearing capacity, were systematically investigated to validate the effectiveness of the “U-shaped ferrule joint bars connections”. The test results indicated that the performance of the “U-shaped ferrule joint bars connections” is reliable. During the loading process, the prefabricated utility tunnel experienced three stages, i.e., cracking, stiffness degradation, and ultimate failure. The numerical analysis results correlated with the test results well. The simulation results showed that the bearing capacities of the prefabricated underground utility tunnel and the cast-in-place utility tunnel were similar. The longitudinal joint connections of the prefabricated utility tunnel allow the structure as an integration to maintain favourable mechanical properties.

## 1. Introduction

An underground utility tunnel refers to an underground tunnel space where municipal pipelines, e.g., electric transmission lines [1], communication systems, and gas and water supply systems, are stored in it. The underground utility tunnel is a lifeline project implemented to ensure the operation of the city. This utility tunnel has become one of the significant symbols of modernized and scientifically managed novel urban municipal infrastructure in the 21st century [2,3]. The underground utility tunnel can effectively solve the problems of dense overhead line networks, repeated road excavation, and frequent pipeline accidents, which is conducive to improving urban functions, beautifying the urban landscape, and promoting intensive and efficient urban development [4,5,6]. Compared with cast-in-place underground utility tunnels, prefabricated underground utility tunnels have the advantages of high production efficiency, high quality, short construction cycle, low labour costs, and environmental friendliness [7,8]. With the industry’s urgent demand for efficient and green constructive technologies, the construction and development of prefabricated urban utility tunnels are actively encouraged.

Since there are plenty of seams and joints in the prefabricated utility tunnel, it is crucial to make a solid connection between the prefabricated components to ensure the structural integrity, bearing capacity, seismic resistance, and the ability to guard against possible water infiltrations. At present, a series of related studies has been conducted on the connection technology of prefabricated utility tunnels. Garg et al. [9] conducted an experimental study on the shear performance of a whole cabin prefabricated utility tunnel, showing that this type of tunnel has a high shear bearing capacity. Fang et al. [10] carried out a full-scale model test of a double-cabin prefabricated utility tunnel and found that the failure of the tunnel was shear failure, which indicates that the shear bearing capacity is a controlling factor in the design of the tunnel. Hu et al. [11] studied a prefabricated slotted section-assembled utility tunnel. They investigated the waterproof performance and mechanical properties of the joints of a prefabricated single-cabin utility tunnel. Moreover, the waterproof performance and mechanical properties were also investigated by Jiang and Tian [12] on a prefabricated utility tunnel structure constructed with “restrained steel plug ring technology” and “restraint lap steel technology”. Wei et al. [13] experimentally investigated the seismic resistance of the joints of a laminated prefabricated underground utility tunnel. Huang [14] numerically analysed the influence law of rigid and flexible joints, and different forms of blocking on the overall seismic resistance of a prefabricated utility tunnel. Yi et al. [15] found that the structural properties of a utility tunnel without axillary corners can be made comparable to those of a utility tunnel with axillary corners by increasing the reinforcement rate of the longitudinal bars in the top plate. Meanwhile, there were new methods, e.g., applications of ultrasonic detection of the quality of steel fibre-reinforced concrete, applied in tunnels [16].

To greatly accelerate the construction and make the construction process more environmentally friendly for the utility tunnel, we proposed the U-shaped ferrule joint bar connections. Based on the technology of U-shaped ferrule joint bar connections, this paper proposes a new type of prefabricated underground utility tunnel structure. We conducted a static test on the prefabricated utility tunnel to obtain its crack development, load-midspan deflection curves, and bearing capacity. We also carried out the corresponding ABAQUS finite element numerical simulations to verify the validity of the model. By comparing crack distribution, load-midspan deflection curves, and the ultimate load of the prefabricated utility tunnel and cast-in-place utility tunnel, we found that the mechanical properties of the prefabricated utility tunnel were comparable to those of the cast-in-place utility tunnel. In addition, we compared the ultimate load of a double-section prefabricated utility tunnel with that of a double-section cast-in-place utility tunnel to investigate the effect of longitudinal connections on the mechanical properties of this proposed tunnel.

## 2. New Type of Prefabricated Underground Utility Tunnel

**Figure 1:**
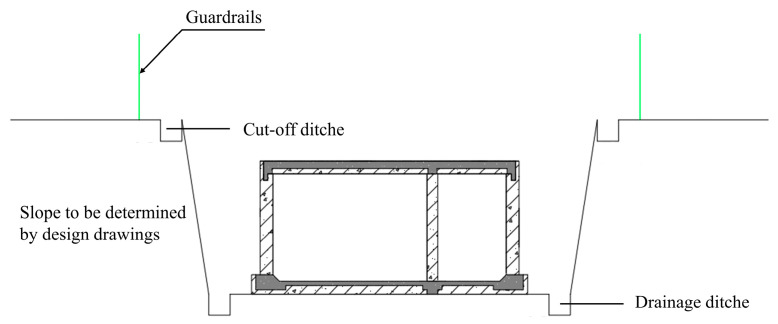
Construction of underground utility tunnel using cut and cover method.

**Figure 2:**
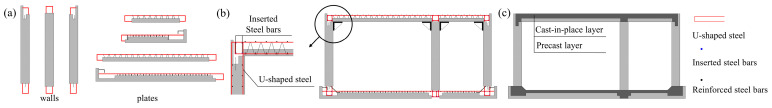
Construction of the new type of prefabricated underground utility tunnel: (**a**) step 1: prefabrication of specimen; (**b**) step 2: assembly of precast components; and (**c**) step 3: concrete pouring of the laminated layer and joints.

**Figure A1:**
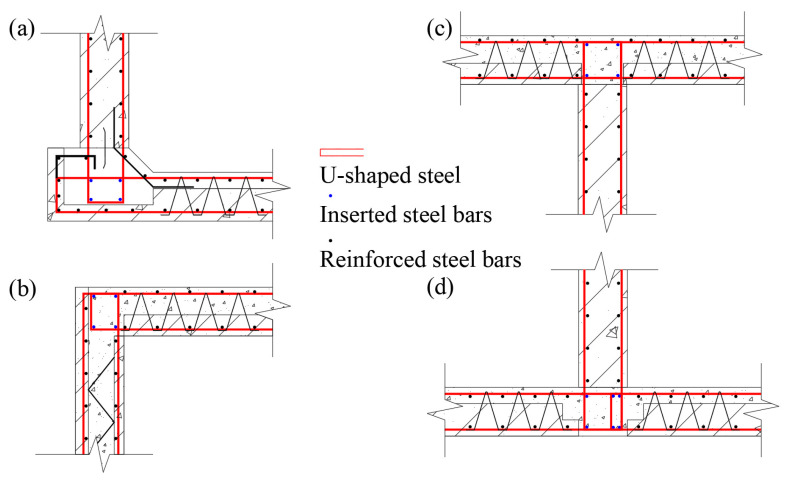
Four kinds of joint connections: (**a**) between lateral wall and top plate; (**b**) between lateral wall and bottom plate; (**c**) between middle wall and top plate; and (**d**) between middle wall and bottom plate.

In consideration of a large cross-section, a shallow buried depth, and construction conditions, the cut and cover method is generally adopted for the construction of urban underground utility tunnels (Figure 1). The top and bottom plates of the prefabricated underground utility tunnel are precast laminated plates and both the lateral and middle walls are fully precasted components. The exposed U-shaped ferrules of each prefabricated component are shown in Figure 2a. When the test specimens are assembled, the U-shaped ferrules at the joints are lapped to each other to form a plane rectangular and longitudinal reinforcement is inserted at the four corners of the plane rectangle (Figure 2b). Then, the laminated plates and joints are poured with concrete to form the prefabricated underground utility tunnel structure, as shown in Figure 2c. There are four kinds of joints in a prefabricated underground utility tunnel in one section (Figure A1). Bolt holes are reserved in the precast walls and steel temporary supporting brackets are installed to construct the roof of the prefabricated utility tunnel without vertical support. The top plate is a prefabricated laminated plate that realizes construction without formwork and greatly improves the construction efficiency. Moreover, the prefabricated counterparts comply with the requirements of green constructions.

## 3. Experimental Study on the Mechanical Properties of Prefabricated Underground Utility Tunnels

### 3.1. Specimen Fabrication

**Figure 3:**
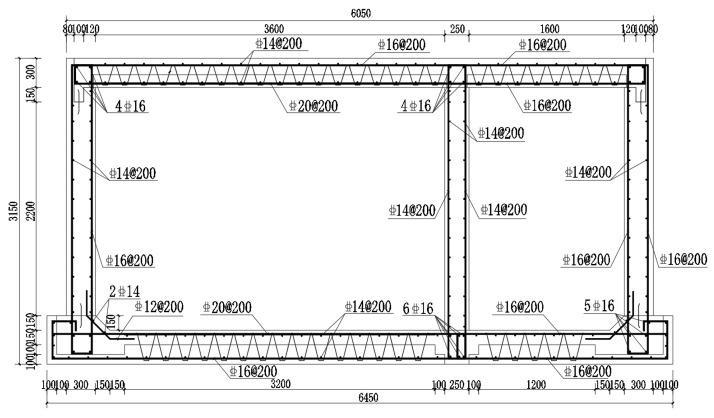
Reinforcement figure of full-scale tunnel model (Units: mm).

The XC2-163625A3S double-cabin utility tunnel in the National Building Standard Design Collection “Cast-in-place Concrete Utility Tunnel” (17GL201) was selected for the study. The test of the prefabricated underground utility tunnel was conducted on a double-cabin full-scale model. Figure 3 shows the dimensions and reinforced bars of the full-scale tunnel model. The net widths of the two cabins were 3600 and 1600 mm, respectively, and the net height of the cabins was 2500 mm. The top plate and its precast layer were 300 and 130 mm thick, and the bottom plate and its precast layer were 350 and 200 mm thick, respectively. The thickness of the lateral wall and the middle wall were 300 and 250 mm, respectively. To facilitate transport and lifting in urban areas, the longitudinal length of the utility tunnel was designed to be 1500 mm. The strength of the precast concrete was C35. To better prevent cracks from forming weak areas between the old and new concrete, the cast-in-place concrete was set as C40. The thickness of the protective layer in the internal side of the precast specimen was 30 mm and that in the external side was 50 mm. According to the test methods in the corresponding standard [17], the actual measured cubic compressive strength of the precast concrete was 45.81 N/mm^2^, while that of the cast-in-place concrete was 45.30 N/mm^2^. All reinforcement types were HRB400 and the average measured strengths of the reinforcement are provided in Table 1.

### 3.2. Test Loading

**Figure 4:**
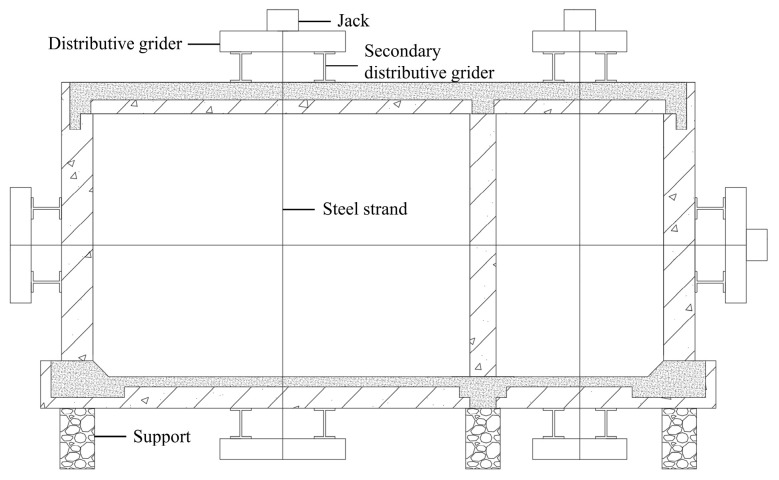
Test loading devices.

**Figure 5:**
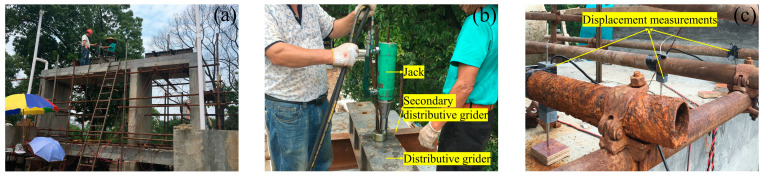
On-site loading of the prefabricated utility tunnel: (**a**) full-scale utility tunnel; (**b**) jack; and (**c**) displacement measurements.

**Figure 6:**
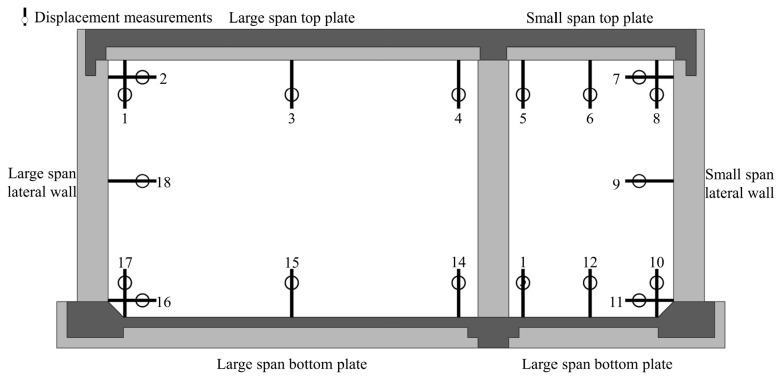
Distribution of displacement measurement points.

During the test, in accordance with the principle of load equivalence, the model was loaded simultaneously by three piercing jacks. The loads were applied by steel strands and secondary distribution girders (Figure 4). The test in site is shown in Figure 5. The test loading procedure is listed in Table 2. First, the model was loaded to the standard value in five steps. Afterwards, in two steps, the load increased to the design load value and, finally, in eight steps, the load increased to steel strand failure. The deflections of the midspan of the top plates, bottom plates, and lateral walls were measured by displacement measurements simultaneously (Figure 6). To ensure the accuracy of the measurement data, these displacement measurements were fixed to a pre-erected scaffold that was not in touch with the full-scale specimen.

### 3.3. Test Results and Analyses of the Prefabricated Underground Utility Tunnel

#### 3.3.1. Crack Development Process

**Figure 7:**
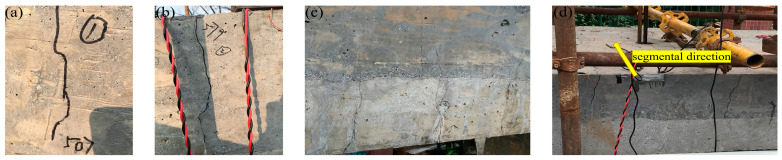
Development of cracks at the beginning of the test: (**a**) the first crack when the load was increased to 507 kN; (**b**) the crack when loaded to 579 kN; (**c**) cracks in midspan tensile zone when the load reached 650 kN; and (**d**) cracks in bottom plate developing along segments when loaded to 650 kN.

When the large-span plate load reached 435 kN, the small-span plate and lateral-wall loads were 196 kN and 289 kN, respectively (Table 2). There was no crack in the structure of the prefabricated utility tunnel. The following description of the crack development in the full-scale structure is based on the loads applied to the large-span plate. When the load was increased to 507 kN, the first flexural crack appeared in the tensile area of the large-span top plate at 400 mm away from the middle-wall support. At a load of 579 kN, two cracks, with a length of approximately 150 mm, appeared in the tensile zone near the middle-wall support and the lateral-wall support of the large-span top plate. In addition, several microcracks appeared in the tensile zone in the middle of the large-span top plate. When the load reached 650 kN, two microcracks appeared in the tensioned area in the span of the large-span bottom plate. Additionally, two cracks appeared in each of the large-span and small-span lateral walls near the top plate support, and the cracks expanded along the segmental direction. The cracks developed at the beginning of the test, as shown in Figure 7.

**Figure 8:**
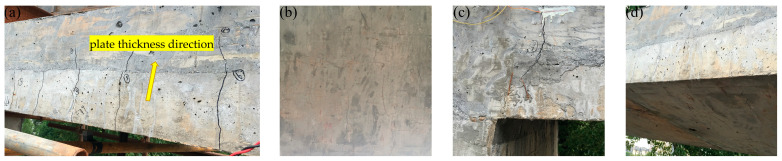
Development of cracks at the end of the test: (**a**) cracks in top plate developing along thickness direction when the load was increased to 722 kN; (**b**) long cracks when the load reached 794 kN; (**c**) a main crack in midspan tensile zone when loaded to 848 kN; and (**d**) cracks in top plate after test was stopped.

**Figure 9:**
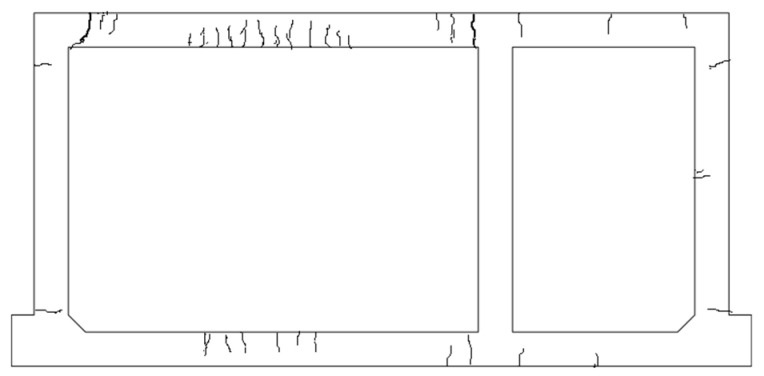
Crack profile of the full-scale model.

When the load was increased to 722 kN, a microcrack appeared in each small-span top plate near the middle-wall support and lateral-wall support. There was one crack in the tensile areas of the large-span bottom plate near the middle-wall support. In addition, the cracks in the large-span top plate near both supports developed along the segmental direction and the cracks in the span developed in the laminated layer along the plate thickness direction. When the load reached 794 kN, a microcrack appeared in the middle of the small-span top plate. A microcrack appeared in both the middle-wall support and middle of the small-span bottom plate. A crack appeared in each of the large-span and small-span lateral walls near the bottom plate support, and several long cracks appeared in the middle of the large-span top plate along the segmental direction. At a load of 848 kN, the cracks in the supports on both sides of the top plate of the large-span plate developed into two main cracks, with an expanding width. The deflection of the top plate suddenly increased. Thus, it could be preliminarily judged that the structure of the prefabricated underground utility tunnel yielded when the span deflection of the large-span top plate was 9.015 mm. When the loading reached the 15th load grade, the steel strand was pulled off and the test was stopped for safety reasons. The final test load value was 940 kN and the deflection of the large-span plate was 12.845 mm. Figure 8 shows the crack development at the end of the test and Figure 9 shows the crack profile of the full-scale model. The cracks were mainly located in the midspan of large-span top and bottom plates, and near the joints, being mainly flexural cracks accompanied by a small amount of flexural/shear diagonal cracks.

#### 3.3.2. Load-Deflection Curve

**Figure 10:**
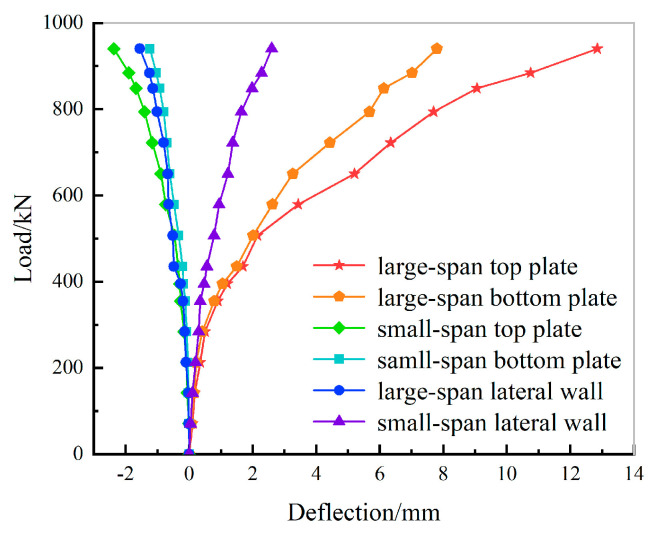
Load-midspan deflection curves.

The load-midspan deflection curves of each lateral wall, top plate, and bottom plate are shown in Figure 10, where the deflection values toward the interior of the cabin were positive and negative towards the exterior. In Figure 10, the load-midspan deflection curves show that the midspan deflection values of the small-span top plate, bottom plate, and large-span lateral wall in the loading process were negative, and the midspan deflection of the middle of the large-span top plate, bottom plate, and small-span lateral wall were positive. When the load increases, the deflection of the large-span top plate increases faster than that of the large-span bottom plate. The deflection of the small-span top plate is faster than that of the small-span bottom plate and the deflection of the small-span lateral wall increases faster than that of the large-span lateral wall. During the loading process, the load-deflection curves of the small-span top plate, small-span bottom plate, small-span lateral wall, and large-span lateral wall tended to be linear at all times. The deflection of the large-span top plate increased rapidly and the deflection increased linearly in the early stages of loading. When the load reached 507 kN, the structure cracked, with stiffness degenerating, and thus the inclination of the load-deflection curve decreased. At a load of 848 kN, the deflection increased faster and the structure was close to yielding. The utility tunnel experienced three stages of cracking, stiffness degradation, and ultimate failure during the loading process.

#### 3.3.3. Analyses of Bearing Capacity Limit States

In the process of structural testing on the prefabricated utility tunnel, the bearing capacity limit state analyses were carried out mainly on the most unfavourable position, i.e., the large-span top plate. The flexural bearing capacity of the large-span top plate was calculated using a double-reinforced rectangular section and both the reinforcement and concrete strength were taken according to the parameters obtained from the material properties test [18]. The calculated flexural bearing capacity of the section according to the Code for Design of Concrete Structures (2010) [19] is provided in Table 3.

According to the limit equilibrium of the tunnel, three plastic hinges were formed in the long-span top plate when the structure experienced bending failure. According to this failure mechanism, the uniform load on the top plate of the tunnel was 298.84 kN/m^2^. The designed load on the top plate of the large span of the utility tunnel was 80.56 kN/m^2^ and the maximum uniform load applied to the top plate of the large span during the test was 174.07 kN/m^2^, which is 2.16 times the load design value. In this case, the utility tunnel structure did not experience a crushing phenomenon and could even continue to bear the loads. The ultimate bearing capacity of the large-span top plate of the utility tunnel is 3.71 times the designed value, which meets the design requirements and has a large safety reserve.

## 4. Numerical Simulations on Prefabricated Underground Utility Tunnels

### 4.1. Simulation of Material Constitutive Relationships and Laminated Surfaces

The steel reinforcement was modelled using the double-folded constitutive model in ABAQUS. For the concrete, the plastic damage constitutive model was used. The stress–strain relationship for the uniaxial compression of concrete was adopted from the constitutive relationship suggested by CEB-FIP (1990) [20]. The uniaxial tension of concrete was adopted from the constitutive relationship suggested in the Code for Design of Concrete Structures (2010) [19] and the material parameters in the concrete plastic damage model were taken as shown in Table 4. The Cullen friction model was used to simulate the relative slip between the precast layer and cast-in-place layer, and the friction coefficient in the penalty friction equation was taken as 0.6 [21].

### 4.2. Establishment of the Finite Element Analysis Model

**Figure 11:**
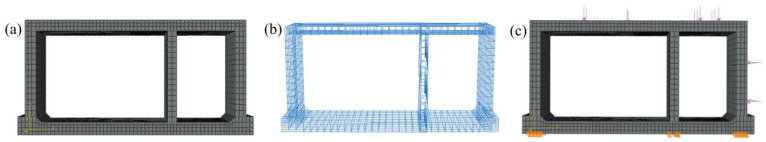
Finite element analysis models of prefabricated underground utility tunnel: (**a**) concrete model; (**b**) reinforcement model; and (**c**) utility tunnel model.

The C3D8R solid elements were used for concrete and the T3D2 truss elements were used for reinforcement. The finite element models of the concrete, reinforcement, and prefabricated underground utility tunnel are shown in Figure 11. The finite element boundary conditions and loading method of the prefabricated underground utility tunnel model were consistent with the test and displacement constraints in the *X*, *Y*, and *Z* directions were applied to the corresponding bottom of the lateral and middle walls of the prefabricated underground utility tunnel.

### 4.3. Finite Element Results and Analysis

#### 4.3.1. Load-Deflection Curves

**Figure 12:**
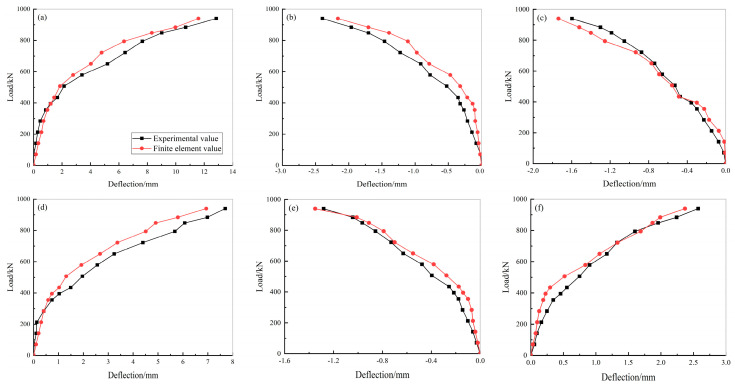
Load-deflection curves of prefabricated underground utility tunnel: (**a**) load-deflection curve of large-span top plate; (**b**) load-deflection curve of small-span top plate; (**c**) load-deflection curve of large-span lateral wall; (**d**) load-deflection curve of large-span bottom plate; (**e**) load-deflection curve of small-span bottom plate; and (**f**) load-deflection curve of small-span lateral wall.

Figure 11 shows the load-deflection curves in the midspan of each lateral wall, top plate. and bottom plate of the prefabricated underground utility tunnel. The finite element analysis values of the maximum deflection are shown in Table 5. Figure 12 and Table 5 show that the calculated load-deflection curve and the test load-deflection curve of the prefabricated underground utility tunnel correlated well, and the calculated values of the maximum deflection in the midspan of the lateral walls, top plates, and bottom plates agreed with the test values, with a maximum error of 9.94%.

#### 4.3.2. Crack Pattern Analyses

**Figure 13:**
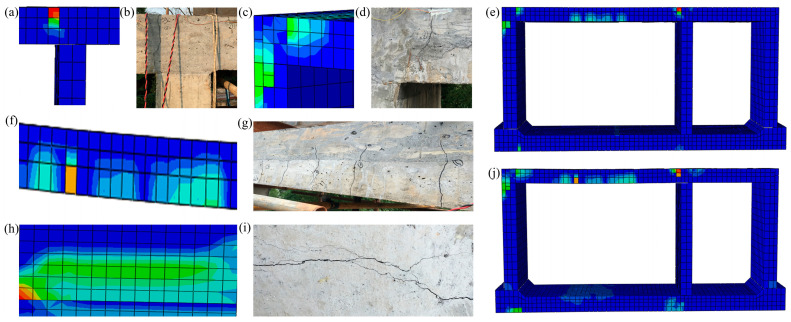
Crack simulation of prefabricated underground utility tunnel: (**a**) the crack near the middle joint for a finite element load of 460 kN; (**b**) the crack near the middle joint for a test load of 579 kN; (**c**) the crack in left joint of large-span top plate at finite element load of 579 kN; (**d**) the crack in left joint of large-span top plate at test load of 579 kN; (**e**) crack distribution at a finite element load of 579 kN; (**f**) through-length cracks along the section direction of large-span top plate with a finite element load of 794 kN; (**g**) through-length cracks along the section direction of large-span top plate with a test load of 794 kN; (**h**) through-length cracks along the section direction near the middle joint for a finite element load of 848 kN; (**i**) through-length cracks along the section direction near the middle joint for a test load of 848 kN; and (**j**) crack distribution at a finite element load of 940 kN.

Plastic strain PEMAG was used to simulate the cracks carried out in the model of the prefabricated underground utility tunnel, which was compared with the cracks in the static tests. When loaded to 460 kN, a vertical crack began to appear near the middle joint of the large-span top plate, as shown in Figure 13a, and when loaded to 579 kN during the test, a crack appeared near the middle joint of the large-span top plate, as shown in Figure 13b. Thus, the finite element analysis crack matched well with the test crack. When loaded to 579 kN, the finite element analysis cracks and test cracks at the left joint of the top plate are shown in Figure 13c,d, respectively. Additionally, cracks appeared in the midspan and joint tension areas of the top plate, as shown in Figure 13e. At a loading of 794 kN, cracks appeared in the midspan of the top and bottom plates of the small span, and cracks in the span of the top plate of the large span extended through the laminated surface with multiple through-length cracks along the section direction, as shown in Figure 13f,g. When the load was increased to 848 kN, the cracks at the supports on both sides of the span top plate developed into through-length cracks along the section direction and the through-length cracks at the supports on the right side of the span top plate are shown in Figure 13h,i. When loaded to 940 kN, each crack continued to develop and the final crack profile of the utility tunnel is shown in Figure 13j. Figure 13 shows the distribution location and pattern of each major crack, and the finite element analysis results agree well with the test results.

### 4.4. Comparative Analysis of Prefabricated and Cast-in-Place Underground Utility Tunnels

#### 4.4.1. Establishment of the Finite Element Analysis Model for the Cast-in-Place Underground Utility Tunnel

**Figure 14:**
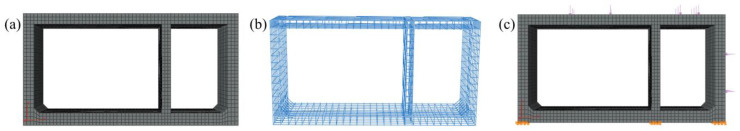
Finite element analysis models of cast-in-place underground utility tunnel: (**a**) concrete model; (**b**) reinforcement model; and (**c**) utility tunnel model.

A finite element analysis model for the cast-in-place underground utility tunnel was established using the same method as for the prefabricated underground utility tunnel, as shown in Figure 14. The same type of graded incremental loading was used until the structure was destroyed.

#### 4.4.2. Load-Deflection Curves

**Figure 15:**
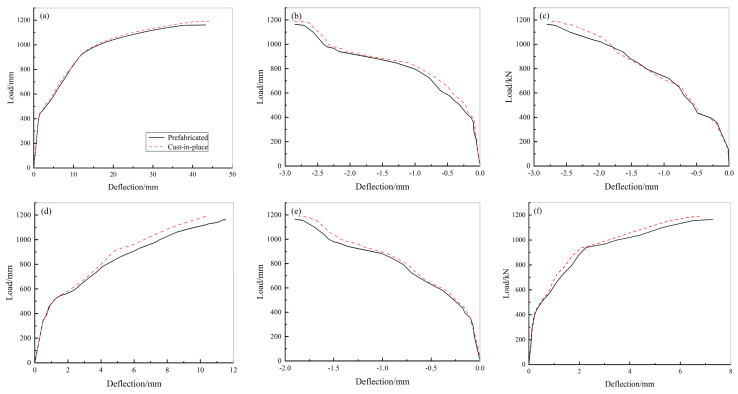
Load-deflection curves of cast-in-place and prefabricated underground utility tunnels: (**a**) load-deflection curve of large-span top plate; (**b**) load-deflection curve of small-span top plate; (**c**) load-deflection curve of large-span lateral wall; (**d**) load-deflection curve of large-span bottom plate; (**e**) load-deflection curve of small-span bottom plate; and (**f**) load-deflection curve of small-span lateral wall.

Figure 15 shows the load-midspan deflection curves of the cast-in-place and prefabricated underground utility tunnels for each lateral wall, top plate, and bottom plate. Figure 15 shows that the load-deflection curves for both the cast-in-place and prefabricated underground utility tunnels are quite similar, indicating that their mechanical properties are basically the same. The ultimate load of the prefabricated underground utility tunnel was 1162 kN and that of the cast-in-place underground utility tunnel was 1191 kN. The ultimate bearing capacity of the prefabricated underground utility tunnel was slightly less than that of the cast-in-place underground utility tunnel.

#### 4.4.3. Crack Analysis

**Figure 16:**
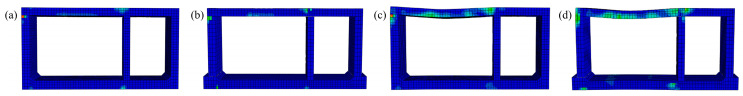
Crack profiles of cast-in-place and prefabricated underground utility tunnels: (**a**) cracking cracks of cast-in-place underground utility tunnel; (**b**) cracking cracks of prefabricated underground utility tunnel; (**c**) failure cracks of cast-in-place underground utility tunnel; and (**d**) failure cracks of prefabricated underground utility tunnel.

Figure 16 shows the crack profiles during the cracking and failure of the cast-in-place and prefabricated underground utility tunnels. Figure 16 shows that cracks appeared successively in the tension area at the middle-wall support as well as midspan and tension zone at the lateral-wall support of the large-span top plate for both cast-in-place and prefabricated underground utility tunnels. Furthermore, the cracks in prefabricated underground utility tunnels appeared slightly earlier than in the cast-in-place utility tunnels. At the time of failure, the cracks in the tensile area at the middle-wall support and in the midspan of the top plate of the cast-in-place and prefabricated underground utility tunnels developed into main cracks with wide crack widths. Throughout the crack development process, it could be seen that the crack distribution pattern of cast-in-place and prefabricated underground utility tunnels was basically the same. However, the crack distribution range of prefabricated underground utility tunnels was slightly larger than that of cast-in-place utility tunnels and the cracking load was slightly smaller.

### 4.5. Analysis of Longitudinal Connection of Sections on the Mechanical Properties of Prefabricated Underground Utility Tunnels

**Figure 17:**
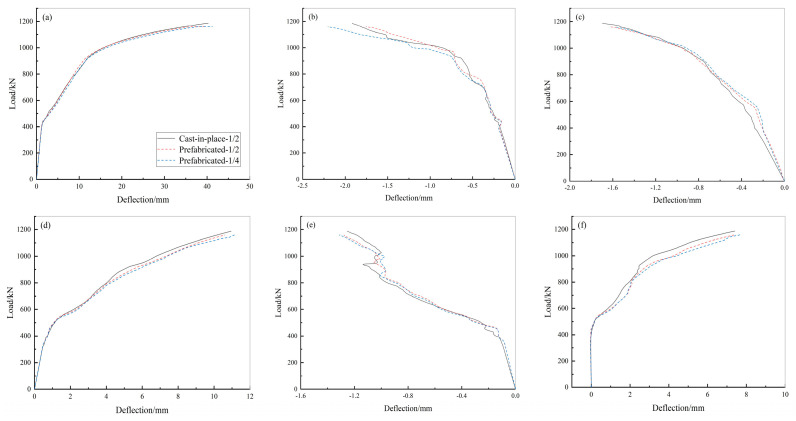
Load-deflection curves of double-section cast-in-place and prefabricated underground utility tunnels: (**a**) load-deflection curve of large-span top plate; (**b**) load-deflection curve of small-span top plate; (**c**) load-deflection curve of large-span lateral wall; (**d**) load-deflection curve of large-span bottom plate; (**e**) load-deflection curve of small-span bottom plate; and (**f**) load-deflection curve of small-span lateral wall.

**Figure A2:**
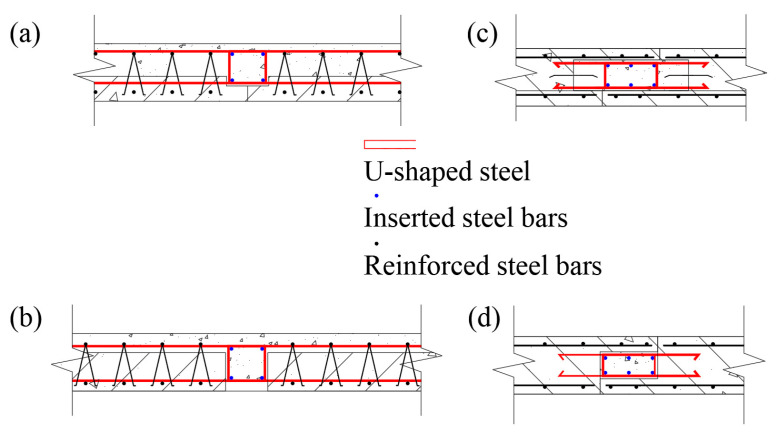
Four kinds of longitudinal connections: (**a**) between bottom plates; (**b**) between top plates; (**c**) between lateral walls; and (**d**) between middle walls.

The longitudinal connections of the sections of the utility tunnel play a significant role in the mechanical properties and security of the structure as, in practical engineering applications, the utility tunnel is constructed from sections that are connected longitudinally to form a solid unit (Figure A2). Following the same method as the previous finite element modelling, a double-section cast-in-place utility tunnel and a double-section prefabricated utility tunnel were modelled. By analysing the ultimate load capacity of both, the effect of the longitudinal connection of the sections on the mechanical properties of the prefabricated utility tunnel was investigated. Figure 17 shows the load-midspan deflection curves for each component of the two kinds of double-section tunnels. The measurement point of the double-section cast-in-place utility tunnel was placed in the longitudinal 1/2 and the measurement points of the double-section prefabricated utility tunnel were placed in the longitudinal 1/2 and 1/4.

Figure 17 illustrates that the load-deflection curves of the measured points of the cast-in-place utility tunnels as well as the longitudinal section connection and non-section connection of the prefabricated utility tunnel are all very similar, which indicates that all of them have similar mechanical properties under the same loading conditions. The ultimate load capacity of the double-section prefabricated underground utility tunnel was 1159 kN and the that of the double-section cast-in-place utility tunnel was 1187 kN. The longitudinal joint connections of the prefabricated utility tunnel allow the structure, as a whole, to maintain favourable mechanical properties.

## 5. Conclusions

Among different steel connection methods, U-shaped ferrule joint bar connections offer more benefits, such as highly precise construction, a relatively simple connection process, and more stable mechanical properties. Therefore, the present study, using these connections, proposed a new type of prefabricated concrete underground utility tunnel. For this purpose, a full-scale model static test and ABAQUS finite element analysis were conducted. Additionally, the mechanical properties, i.e., the crack development law and bearing capacity, were tested for validation. The following conclusions can be drawn from the results.

- The U-shaped ferrule joint bar connections are reliable. When the test is loaded to the tunnel design load value of 435 kN, the prefabricated double-cabin underground utility tunnel structure does not show any cracks. When it is loaded to 507 kN, the first crack appears 400 mm away from the middle-wall support in the top plate of the large span of the underground utility tunnel. When the load increases, the cracks develop continuously and they are mainly concentrated near the support and midspan of the wall or the plate structure of the tunnel.
- The ultimate bearing capacity of the prefabricated double-cabin underground utility tunnel is 3.71 times the load design value, while the ultimate bearing capacity meets the design requirements and has a large safety reserve.
- The load-deflection curves for both the cast-in-place and prefabricated underground utility tunnels are basically the same. The ultimate load of the prefabricated underground utility tunnel is 1162 kN and that of the cast-in-place underground utility tunnel is 1191 kN. The ultimate bearing capacity of the prefabricated underground utility tunnel is slightly less than that of the cast-in-place underground utility tunnel.
- The crack distribution pattern of cast-in-place and prefabricated underground utility tunnels is basically the same. The crack distribution range of prefabricated underground utility tunnels is slightly larger than that of cast-in-place utility tunnels and the cracking load is slightly smaller.
- The load-deflection curves of the measured points of the cast-in-place utility tunnel as well as the longitudinal section connection and non-section connection of the prefabricated utility tunnel are all very similar. The ultimate load capacity of the double-section prefabricated underground utility tunnel is 1159 kN and that of the double-section cast-in-place utility tunnel is 1187 kN. The longitudinal joint connections of the prefabricated utility tunnel allow the structure, as a whole, to maintain favourable mechanical properties.

## Figures and Tables

**Table 1:** Steel strength in the specimens.

Steel Grade	Diameter/mm	Yield Strength *f_y_*/Mpa	Ultimate Strength *f_u_*/Mpa
HRB400	12	428	616
HRB400	14	419	592
HRB400	16	446	622
HRB400	20	453	641

**Table 2:** Test loading procedure.

Loading Grade	Large-Span Plate/kN	Small-Span Plate/kN	Lateral Wall/kN
1	71	32	47
2	142	64	94
3	213	96	141
4	284	128	188
5 (standard value)	355	160	235
6	395	178	262
7 (design value)	435	196	289
8	507	229	336
9	579	261	383
10	650	293	430
11	722	325	478
12	794	358	526
13	848	382	561
14	884	398	585
15	940	424	622

**Table 3:** Bending capacity of the large-span top plate.

Section Position	Tensile Reinforcement/mm^2^	Compressed Reinforcement/mm^2^	Bending Capacity/kN·m
Lateral support	2199	2199	386.5
Midspan	2199	1407	339.7
Middle support	2199	2199	386.5

**Table 4:** Material parameters in the concrete plastic damage model.

*Ѱ* (°)	*ϵ*	*f_b_*_0_/*f_c_*_0_	*K*	*μ*
30	0.1	1.16	0.6667	0.0005

**Table 5:** Midspan deflection value.

Measuring Points	Experimental Value/mm	Finite Element Value/mm	Relative Error
Large-span top plate	12.845	11.593	9.75%
Small-span top plate	−2.396	−2.164	9.68%
Large-span lateral wall	−1.597	−1.737	8.76%
Large-span bottom plate	7.703	6.937	9.94%
Small-span bottom plate	−1.281	−1.350	5.39%
Small-span lateral wall	2.572	2.370	7.85%

## Data Availability

The data provided in this study could be released upon reasonable request.

## Appendix A

There are four kinds of joint connections of a cross-section, i.e., between the lateral wall and top plate, between the lateral wall and bottom plate, between the middle wall and top plate, and between the middle wall and bottom plate (Figure A1).

The longitudinal connections of the prefabricated utility tunnel are mainly divided into four kinds: between bottom plates, between top plates, between lateral walls, and between middle walls, which are shown in Figure A2.
